# The Living Alone with Cognitive Impairment Project’s Policy Advisory Group on Long-Term Services and Supports: Setting a Research Equity Agenda

**DOI:** 10.3390/ijerph19106021

**Published:** 2022-05-16

**Authors:** Elena Portacolone, Jacqueline M. Torres, Julene K. Johnson, Donna Benton, Thomas Rapp, Thi Tran, Paula Martinez, Carrie Graham

**Affiliations:** 1 Institute for Health & Aging, University of California, San Francisco, CA 94158, USA; julene.johnson@ucsf.edu (J.K.J.); thi.tran@ucsf.edu (T.T.); paulam3@uci.edu (P.M.); cgraham@chcs.org (C.G.); 2 Philip Lee Institute for Health Policy Studies, University of California, San Francisco, CA 94158, USA; 3 Department of Epidemiology and Biostatistics, University of California, San Francisco, CA 94158, USA; jacqueline.torres@ucsf.edu; 4 Leonard Davis School of Gerontology, University of Southern California, Los Angeles, CA 90089, USA; benton@usc.edu; 5 LIRAES, Université Paris Cité, 75006 Paris, France; thomas-rapp@u-paris.fr; 6 Sciences Po Paris, LIEPP, 75006 Paris, France; 7 Center for Health Care Strategies, Hamilton, NJ 08619, USA

**Keywords:** living arrangements, health disparities, diagnosis, health care services, long-term services and supports, policy, United States

## Abstract

(1) Background: A United States national policy advisory group (PAG) was convened to identify barriers and facilitators to expand formal long-term services and support (LTSS) for people living alone with cognitive impairment (PLACI), with a focus on equitable access among diverse older adults. The PAG’s insights will inform the research activities of the Living Alone with Cognitive Impairment Project, which is aimed at ensuring the equitable treatment of PLACI. (2) Methods: The PAG identified barriers and facilitators of providing effective and culturally relevant LTSS to PLACI via one-on-one meetings with researchers, followed by professionally facilitated discussions among themselves. (3) Results: The PAG identified three factors that were relevant to providing effective and culturally relevant LTSS to PLACI: (i) better characterization of PLACI, (ii) leveraging the diagnosis of cognitive impairment, and (iii) expanding and enhancing services. For each factor, the PAG identified barriers and facilitators, as well as directions for future research. (4) Conclusions: The barriers and facilitators the PAG identified inform an equity research agenda that will help inform policy change.

## 1. Introduction

An estimated 4.3 million people with cognitive impairment (CI) live alone in the United States (US) [1], which is a population about the same size as Los Angeles. CI includes mild cognitive impairment and Alzheimer’s disease and related dementias. Although the racial/ethnic background of people living alone with CI has not been characterized yet, people of color are likely to be over-represented for two reasons. First, evidence suggests that Black and Latinx older adults (aged 65 and over) are 1.5–2 times more likely to have cognitive impairment than non-Latinx white older adults [2,3,4,5,6]. Second, living alone is common across all racial/ethnic groups: 30% of white older adults live alone, followed by 28% of Black older adults, 20% of Latinx older adults, and 14% of Asian older adults [7,8,9]. Previous studies, primarily of older whites, showed that older adults with CI who live alone, compared to those living with cohabitants, are at higher risk for health threats, including self-neglect [10,11,12,13], malnutrition [12,14], and falls [10,12,14].

Increasing evidence suggests that these negative health outcomes are directly related to a lack of essential long-term services and support (LTSS) provided by family members, friends, or professionals [15,16,17,18,19,20,21,22,23]. In our studies [1,24,25,26,27,28,29], we found that people living alone with cognitive impairment (PLACI) often have limited access to LTSS because they lack cohabitants, who typically provide most (80%) of the unpaid LTSS in the US [18]. Cohabitants are also likely to seek and organize LTSS. Indeed, a major difference between people with CI living alone as opposed to counterparts living with others is the availability of unpaid support from family members and significant others. Limited access to essential LTSS increases the distress of PLACI, as well as costs related to unnecessary hospitalizations and institutionalizations [17,18,19,30].

Using specific formal LTSS (Table 1) is critical to preventing adverse health outcomes among PLACI, who do not have cohabitants to compensate for their CI [15,16,20,21,22,31,32]. In the US, access to formal LTSS avoids unnecessary hospitalizations and institutionalizations, thereby saving on Medicare and Medicaid costs (the government-subsidized healthcare programs that cover most older adults and low-income older adults, respectively) [17,18,19,30,33]. Compared to people with CI living with others, PLACI have an increased need for LTSS due to their diminished capacity for essential but cognitively demanding skills, such as managing medications, procuring groceries, and paying bills [34].

CI also decreases their ability to manage complex medical regimens and makes it difficult to manage major financial decisions [35,36]. The coronavirus (COVID-19) pandemic put a spotlight on the essential role of formal LTSS in supporting older adults living alone with CI [28].

**Figure 1:**
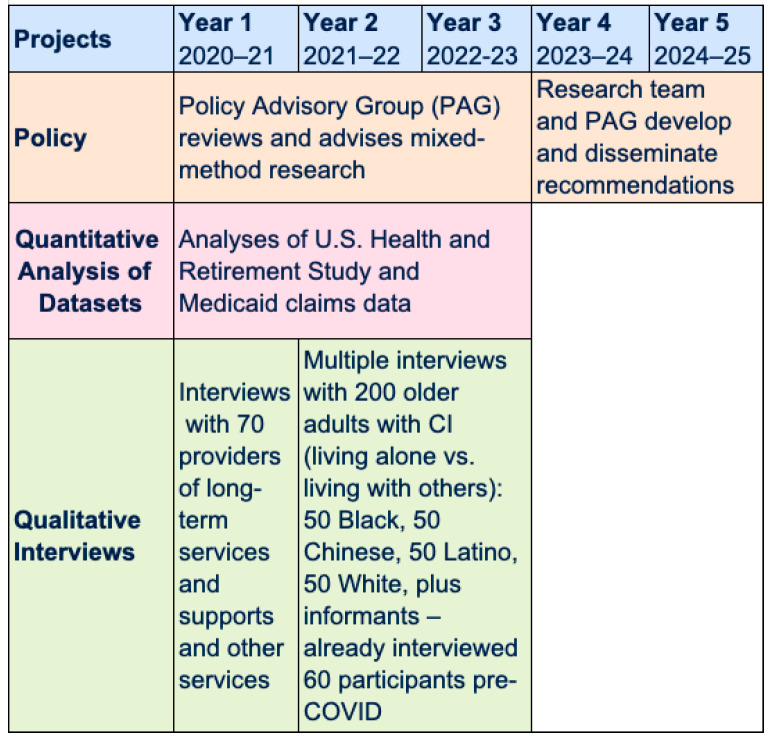
Timeline and activities of the Living Alone with Cognitive Impairment Project.

To identify and address the unmet needs for LTSS for PLACI, in 2020, we launched the “Living Alone with Cognitive Impairment Project.” The goal of this 5-year project is to leverage evidence from mixed-methods research to develop policy recommendations to ensure equitable access to formal LTSS for PLACI (Figure 1). This project focuses on PLACI of color because there is limited understanding of the needs of people of color living alone with CI despite emerging evidence of inadequate access to LTSS, as documented in our studies [1,28]. Furthermore, existing evidence suggests that people of color with CI, whether living alone or with others, are less likely to access and use healthcare services because of delayed diagnosis, misdiagnosis, and challenges in accessing services and referrals to CI-specialized care [37]. Indeed, one study of Black older adults with CI found that a high proportion (65%) of those who lived alone lacked LTSS, but this study did not identify barriers and facilitators to LTSS [34,38]. The project discussed herein is breaking new ground in examining barriers and facilitators to LTSS access for diverse PLACI.

Equity is central to the Living Alone with Cognitive Impairment Project because its overarching purpose is to understand, explain, and reduce the unequal distribution of health and resources across groups [39]. In this project, the criteria used to identify groups are living arrangements and race/ethnicity. Specifically, this project aims to reduce any unequal distribution of health and resources between people with CI living with others versus counterparts living alone, as well as between white people with CI versus their counterparts of color.

As a result, policy recommendations developed by this project aim to ensure that (1) the condition of living alone, as opposed to living with others, does not hamper access to and use of essential services by people with CI, and (2) people of color living alone with CI receive timely and culturally relevant LTSS.

To launch the Living Alone with Cognitive Impairment Project, we convened a Policy Advisory Group (PAG) of recognized experts at the national and state level in LTSS policies for people with CI. The PAG includes 17 policymakers, health service administrators, advocates, and researchers from diverse organizations (see Acknowledgments). The purpose of the PAG is to ensure that the findings of the research team can be used to develop policy recommendations to ensure the equitable access and use of essential formal LTSS for PLACI.

This article presents the research agenda identified by the PAG that informs the ongoing mixed-methods research. This research equity agenda informs the investigations of the PLACI project. Furthermore, this agenda can be useful to the overall scientific community to expand our understanding of PLACI.

## 2. Materials and Methods

The first step in the Living Alone with Cognitive Impairment Project was to elicit the perspective of PAG members on factors hampering and facilitating access to LTSS by PLACI. For each barrier and facilitator, we also elicited directions for a research agenda aimed at expanding our understanding of these barriers and facilitators. Over 12 months (October 2020–September 2021), PAG members first discussed, for one hour each, their research ideas and suggestions individually with the first and last authors over video conference. Specifically, PAG members received a summary of the goals of the Living Alone with Cognitive Impairment Project by email, and during the meeting, they were asked about the major barriers and facilitators for PLACI to access LTSS, with an emphasis on PLACI of color. PAG members then convened in a full 90-minute meeting, where the summary of the research agenda was discussed. The individual conversations and the full meeting occurred via video conference and were recorded and professionally transcribed. Key topics, as well as related barriers and facilitators, of a preliminary equity agenda were then identified from the proceedings of these meetings. Specifically, transcripts of meetings were loaded into ATLAS.ti for a qualitative data analysis that used collaborative deductive content analyses [40,41]. Transcripts of meetings were analyzed line by line by the first author to identify specific barriers and facilitators. A code was created every time a particular barrier or facilitator was identified. A senior coder reviewed the first author’s codes until interpretative convergence was achieved. Definitions of codes and related categories were documented in a codebook and shared with the research team. Next, other independent coders coded the rest of the transcripts; each coded transcript was reviewed by a second coder to achieve interpretative convergence. Additional codes were added with the approval of the research team. Saturated themes were then identified by the first author by making connections between codes, writing memos, and having iterative discussions with the research team. These themes and related barriers and facilitators were then reviewed, refined, and finally approved by PAG members via email communications, and are presented here.

## 3. Results

The PAG identified three factors that are relevant to providing effective and culturally relevant LTSS to PLACI: (1) better characterization of PLACI; (2) leveraging the diagnosis of cognitive impairment; and (3) expanding and enhancing services. For each factor, the PAG identified barriers and facilitators, as well as directions for future research and policies (see Table 2).

### 3.1. Better Characterizing PLACI

#### 3.1.1. Limited Understanding of PLACI

One barrier to developing appropriate services for PLACI includes the lack of systems to identify these individuals or to characterize this group, both in terms of both commonalities and within-group differences. Tremendous heterogeneity exists among this group with respect to the stage of CI, dementia subtype, and demographic and social network characteristics, yet this heterogeneity has not been fully described or recognized. Regarding directions for research, in order to inform policy changes, researchers need to gather evidence on the numbers and characteristics of PLACI by providing replicable tools to track PLACI. With regard to quantitative research, one potential challenge to characterizing via large national databases such as the Health and Retirement Survey (HRS) is that study participants whose CI prevents them from participating directly in the survey may be less likely to identify reliable proxy informants if study participants live alone. As a result, people who had lost the cognitive ability to participate in surveys and live alone may be more likely to be excluded from such surveys, although this point deserves further investigation. As a result, the estimated 4.3 million PLACI identified by our group may be underestimated. In addition, PLACI may be more likely to be attritors in large panel surveys, like the HRS. When such attrition is not exogenous, attrition can raise selection issues and bias the results of empirical studies. Consequently, administrators should document in detail the reasons why PLACI should participate or leave large surveys. On a related note, research should expand the body of knowledge on different typologies of PLACI, with particular attention to race/ethnicity, immigration status, languages spoken, religious affiliation, and sexual orientation, as well as diagnosis status. It is also important to assess the presence and characteristics of caregivers as well as flag the absence of appropriate caregivers. Furthermore, in order to ensure timely access to effective LTSS, researchers should identify and better understand subcategories of PLACI who may be particularly at risk, such as victims of life-long discrimination, recent widow(er)s, and those with a history of depression and/or suicidal ideations [26,29].

#### 3.1.2. Understanding Specific Needs of PLACI

In order to recommend policies, it is essential to have a comprehensive understanding of the specific needs of PLACI with regard to their physical, cognitive, mental, and emotional health. The PAG raised concerns about the fact that current public services partially catered to physical needs only, mostly with regard to physical disabilities and keeping the house clean, while failing to consider cognitive, mental, and emotional needs. Data on unmet needs are important for ascertaining whether policymakers should consider increasing coverage for LTSS via Medicare or the Older Americans Act. In order to inform policy changes, researchers need to elucidate the range of services that PLACI needs and whether existing integrated delivery systems could better serve the specific needs of PLACI.

### 3.2. Leveraging the Diagnosis of CI

#### 3.2.1. Unintended Adverse Consequences of the Diagnosis of CI

A known barrier to seeking a diagnosis of CI is the limited benefits stemming from it. Receiving a diagnosis of CI is associated with a loss of privileges (e.g., losing a driver’s license) and status (e.g., being considered incompetent). Its process can be embarrassing and exhausting, while providing few payoffs. Although a diagnosis may lead to being prescribed medicines (of limited benefit), a CI diagnosis does not result in qualification for LTSS. What is more, a diagnosis of CI or a prescription of pharmaceuticals to delay CI (e.g., Aricept) will often disqualify a person for admittance into an assisted living facility or a continuous care retirement community. In some senior communities, a diagnosis of CI might lead to eviction. Furthermore, individuals with a diagnosis of CI cannot qualify to purchase long-term care insurance and may face hampered access to mental health services [29]. To inform policy changes, researchers need to better understand the effects of receiving a diagnosis by race/ethnicity and from patients’ and providers’ perspectives, with particular attention to race/ethnicity and overall equity. It is also pressing to understand whether PLACI are less likely than those living with others to receive a diagnosis of CI, especially considering that more than 50% of people with CI are undiagnosed [42]. This percentage seems likely to be higher in PLACI because they lack cohabitants who would notice the symptoms of their CI.

#### 3.2.2. Learning How to Empower PLACI at the Point of Diagnosis and Beyond

The PAG members suggested offering a “wrap-around” set of services at the point of diagnosis that provides instrumental and emotional support to PLACI to empower them. For example, the shock of losing a driver’s license could be mitigated by receiving vouchers for free taxi rides or comparable transportation. Expedited access to qualified home care aides would also help. The benefits of a wrap-around set of services would include increasing the appeal of receiving a diagnosis of CI. In addition, this intervention might decrease hospitalizations, with related savings for Medicare and Medicaid. To facilitate policy changes, researchers need to understand what items (e.g., LTSS, technologies) are more beneficial to PLACI and what types of emotional and empathic support would be more welcome and culturally sensitive. International comparisons would be useful to understand what LTSS are provided elsewhere at the point of diagnosis, as well as their effects.

### 3.3. Expanding and Enhancing Services

#### 3.3.1. Learning How to Elevate the Status of Home Care Aides

Home care aides provide LTSS that are essential to PLACI because, when living alone with CI, it is important to receive consistent daily help with activities such as procuring groceries, cooking food, and keeping up with appointments. Home care aides can provide these essential services while also providing emotional support, which decreases isolation. In particular, the ongoing pandemic pointed to the essential role of home care aides in supporting PLACI [28]. To ensure access to qualified, affordable, language-concordant home care aides, expanding the criteria to access publicly paid home care aides is the first step. A major barrier to providing effective LTSS is that Medicare does not pay for most personal assistance services. Medicare coverage is only available if services are combined with skilled nursing, therapy, and rehabilitation services. The cost of a paid personal care assistant can be quite unaffordable for those who do not cohabitate with a caregiver or have family members providing unpaid care, or who do not qualify for Medicaid in states with sufficient coverage for home- and community-based services. Indeed, paying out-of-pocket for a home care aide for daily care can cost more than $4000 per month [43]. Round-the-clock home care could exceed $200,000 per year. For those who can purchase long-term care insurance, the yearly premiums can be upward of $3000 per year. A second step to expanding access to home care aides is to elevate the status of the profession of home care aides by better understanding the role played by education opportunities, equitable compensations, and career advancements built within this profession. To facilitate policy changes, researchers need to provide evidence of the effect of the presence of home care aides on PLACI’s wellbeing. Researchers also need to better understand the priorities and concerns of public and private home care aides, as well as their employers’ perspectives. It is also important to understand what mechanisms need to be in place to increase the status of this profession with increased pay, training, and career advancements. Furthermore, it is important to better understand the most appropriate supports and services are for family members and other caregivers to ensure that they are adequately paid for. It is also pressing to better understand how to honor immigrants providing LTSS [44]. International comparisons will also be beneficial to understanding how to increase the standing of home care aides, as well as how to properly compensate them and monitor caregivers providing home care. For example, in Denmark, the home care aide position offers career advancements, and caregivers who are paid to provide home care are closely monitored [27].

#### 3.3.2. Restrictive Income Eligibility Criteria to Access Medicaid Home- and Community-Based Services (HCBS)

A major barrier preventing PLACI from accessing Medicaid HCBS services is income thresholds that allow only PLACI with extremely low incomes to qualify. Although all states have at least one Medicaid waiver program that provides select home care services, almost all states have waiting lists for these programs [45]. As a result, most PLACI are ineligible for government-subsidized home care aides, which are also not covered by Medicare. To make things worse, a related practice that is common in the US is the “spending down” of assets to qualify for Medicaid. This practice is especially detrimental to PLACI of color because it hampers the transfer of generational wealth, thereby making it more difficult to escape poverty. In addition, Medicaid estate recovery provisions allow states to recover prior costs of care by taking over trusts and real estate properties of deceased beneficiaries, further reducing assets transferred to younger generations. To inform policy changes, it is important that researchers quantify this public health issue by estimating the number of PLACI who do not qualify for Medicaid or qualify only after spending down, as well as funds lost to families via the Medicaid estate recovery program. Qualitative research can illuminate the effects of these policies.

#### 3.3.3. Adapting to Diverse Cultural Values

Making culturally appropriate services available seems likely to increase the acceptance and use of LTSS. It is essential to pay attention to specific preferences and cultural patterns deriving from diverse immigration histories, language spoken, citizenship status, and history of trauma and discrimination. Individual traits (e.g., introversion) are also likely to play a role in considering a diagnosis. Some cultures may also believe that the family plays a more prominent role than the individual [46,47]. It might be important to pay attention to sexual orientation given that older LGBTQ adults may be likely to live alone by not having had spouses or children. To support policy recommendations, researchers need to understand the features of appropriate care from PLACI’s perspective.

#### 3.3.4. Learning How to Have a Unique Integrated Point of Reference to Access Services

It is generally difficult for PLACI to access appropriate services because the information is not widely available and easy to find. Furthermore, services are usually “siloed” from one another into different departments (e.g., federal, state), which facilitates this fragmentation. In addition, shortages of home-based LTSS workers are prominent in the US [48]. Most PLACI access few services until some “catastrophe” (e.g., falls, fires) occurs that might connect them to additional services. PLACI of color might have more difficulties in locating services or being aware of the existence of services due to systemic biases and barriers to care driven by systemic racism and limited language-concordant healthcare providers. In order to develop specific policy recommendations, it is important to better understand the feasibility of providing a one-stop point of service for all services related to the care of PLACI. At this point of service, people with CI and caregivers (if available) would receive education, information, and support in accessing services, including home care aides and licensing to become a family home care aide. To facilitate policy changes, researchers need to understand whether and how innovative initiatives to provide one-stop points of service can be tailored to PLACI [49]. For example, in the US, a promising initiative that increased collaboration between providers is the comprehensive dementia care management program at the University of California, Los Angeles, which relies on nurse practitioners working as dementia care managers and focuses on people with cognitive impairment with caregivers [49]. Another innovative initiative is the Washington state public long-term care insurance program, which gives taxpayers the option to contribute to a fund that will take care of their LTSS needs for up to $36,500 for the first year of needed LTSS [50]. It is important that researchers understand how innovative initiatives can be leveraged to allocate resources toward PLACI.

#### 3.3.5. Learning How to Incorporate Technology into PLACI’s LTSS

Given the increase in telehealth initiatives prompted by the COVID-19 pandemic, it is essential to understand which technologies have the potential to empower and support PLACI, with the likelihood that they need extra support to use these technologies. Specifically, it is important to understand what technologies could be used at the point of diagnosis and what type of support is needed. To identify these technologies, future research could draw lessons from the successful use of telehealth to manage other chronic conditions among older adults, such as diabetes [51] and cancer [52]. Furthermore, addressing ethical issues that might arise when offering telehealth to PLACI is critical, with attention to consent and monitoring [53]. It is also important to understand how and whether the perspectives of PLACI differ according to their race/ethnicity and other characteristics. Finally, it is crucial to understand how to increase LTSS workers’ skills related to using technologies [48] to support PLACI while respecting their values.

## 4. Discussion

The involvement of the PAG from the very beginning of the project has ensured that both quantitative and qualitative portions of the investigations address gaps in knowledge that can be used as evidence for policy recommendations to expand the access of essential LTSS to PLACI. This policy-driven approach is critical because the final outcome of the Living Alone with Cognitive Impairment Project is the development and widespread dissemination of these evidence-based policy recommendations in the last two years of the project, as illustrated in Figure 1. To support the development of sound policy recommendations, the investigation will compare and contrast PLACI versus people with cognitive impairment living with others as much as possible because comparisons add depth to the data.

In this first phase of the project, the directions of the PAG informed the research plan of the PLACI project by identifying priorities, as well as opportunities for both quantitative and qualitative portions of the project.

With regard to the quantitative investigation, we will prioritize further characterizing PLACI and any unmet needs via comparisons of PLACI versus people with cognitive impairment living with others. As a first step, we will better understand whether any differences emerge between people with cognitive impairment living alone as opposed to those living with others with regards to difficulties with carrying out essential activities of daily living (e.g., toileting, washing themselves) and instrumental activities of daily living (e.g., buying groceries, paying bills) with and without help received by family or non-family caregivers. To further characterize PLACI, we will evaluate whether age, gender, race/ethnicity, and rural (vs. urban) residence modifies the association between living alone and the difficulty of carrying out instrumental activities of daily living (I/ADL) without help. Furthermore, because home care aides are critical to the PLACI’s wellbeing, the quantitative investigation will prioritize identifying gaps in accessing home care services, building on prior evidence suggesting that, in the US, PLACI have limited access to home care aides [34].

With regard to the qualitative portion, we will conduct in-depth interviews with providers of services to people with CI to identify specific unmet needs of those living alone as opposed to counterparts living with others. Providers will include physicians, nurses, social workers, home care aides, case managers, and adult-day-center managers. We will also elicit providers’ perspectives on any differences in services for PLACI vs. counterparts living with others. The comparison will illuminate the potential role of living arrangements in facilitating or hampering access to services for people with CI. These interviews will further elicit strategies to identify PLACI, as well as identify and address unmet needs. Because of the wide range of providers included in the investigation, data will encompass wide perspectives. Interviews with providers will also be used to better understand how to empower PLACI at the point of the diagnosis and gather recommendations, as well as best practices, on ways to elevate the status of home care aides, adapt to diverse cultural values, and leverage telehealth. Next, people with CI living alone and living with others from diverse racial/ethnic backgrounds and speaking either English, Spanish, or Cantonese, including individuals living alone and those living with others, will be interviewed multiple times to understand directly from them what matters to them and what barriers they encounter to access essential services. Once again, comparing two groups of participants with cognitive impairment (i.e., living alone vs. living with others) will enable us to better understand the potential role played by living arrangements in facilitating or hampering access to services.

While these investigations are underway, the research team also aims to disseminate findings to policymakers involved in allocating resources to vulnerable populations. Specifically, due to the policy experts’ involvement in the design of the investigation, we are learning to frame findings via policy briefs because these documents are consulted more often than academic papers by policymakers. The involvement of policy experts also makes the research team sensitive to short- and long-term opportunities to potentially expand services to PLACI. For example, in the short term, a program that can be leveraged to allocate resources toward PLACI is the American Rescue Plan Act, through which, the US federal government has transferred $1.9 trillion to US states to support residents to mitigate the effects of the COVID-19 pandemic. The injection of these funds is now forcing state officials to decide on the optional allocation of these funds. In the long term, a policy recommendation under consideration includes the expansion of Medicare with new flexibilities to pay for non-medical services by leveraging additional payment models that cover additional resources.

Finally, to gain a broad perspective on comprehensive policies and programs providing LTSS to PLACI, scholars of PLACI from multiple disciplines (e.g., economy, policy, social sciences) were invited to join an international advisory group fostering international comparisons between PLACI residing in the US as opposed to those residing abroad by drawing from quantitative, qualitative, and mixed methods. The purpose of these comparisons is to identify socially constructed (thus, addressable) barriers and facilitators to access essential LTSS.

Limitations of the qualitative portion of the Living Alone with Cognitive Impairment project include conducting qualitative research in only two states (i.e., California and Michigan) in the US and focusing on only four racial/ethnic groups (i.e., Black, Chinese, Latino/Hispanic, and white older adults). Future studies of our group will expand to other races and ethnicities (e.g., Native Americans, South Asians). Limitations of the quantitative portion of the study include using databases that could underrepresent PLACI. In addition, HRS has limited data on racial/ethnic subgroups beyond Black, Latinx and white older adults (e.g., Asian American or Native American older adults).

Findings emphasize the importance of researchers and policymakers closely collaborating from the onset of investigations in order to efficiently and rigorously translate research findings into policy recommendations. Findings also underscore the importance of eliciting the perspectives of providers to learn from their experience serving PLACI. Furthermore, providers can provide insightful recommendations on how to expand LTSS to PLACI, as well as how to identify PLACI and support them. An implication of this study for providers is to pay particular attention (if not already done) to patients or clients who live alone and have cognitive impairment. Special attention should be devoted to identifying PLACI who are lonely or at risk. According to the profession, this attention could translate into extra home visits, follow-ups, resources, combined therapies, and connections with local resources to foster a sense of belonging [54].

## 5. Conclusions

A large PLACI population in the US has limited access to essential services. To date, PLACI are seldom mentioned in plans to support people with LTSS at the national and state levels. The lack of formal LTSS has negative repercussions on the mental and psychological health of PLACI, as well as their ability to remain living in the community [24,25,27,55]. Furthermore, the lack of formal LTSS places disproportionate burdens on unpaid caregivers [56], who are often women of working age [57]. In order to address this public health crisis, it is important to further elucidate specific barriers and facilitators to provide appropriate, affordable, and culturally relevant services to this population. A rigorous understanding of these barriers and facilitators will support the development of policy recommendations to expand the allocation of resources toward PLACI and LTSS providers. The need for policies fully supporting PLACI points to the critical responsibility of researchers to work closely with policy experts.

## Figures and Tables

**Table 1:** Key formal long-term services and support (LTSS) providers for PLACI and examples of support.

LTSS	Examples of Support
Home-care aides	House cleaning, buying/cooking food, reminders, errands, dressing, bathing, transportation, cognitive stimulation
Support coordinators (e.g., social workers, nurses, case managers)	Service coordination, needs assessments, managing safety, as well as legal, financial and housing matters
Adult day healthcare centers	Medical services, service coordination, safety, finances, cognitive stimulation
Meal services	Nutrition
In-home medical services (e.g., medical providers)	Managing medications, health assessment and monitoring
Transportation services	Transportation to services (e.g., medical, social, personal)

**Table 2:** Overview of results.

Factors and Sub-Sections	Barriers (B) and Facilitators (F)	Research Directions	Policy Directions
**1. Better characterizing PLACI**			
Limited understanding of PLACI	- B. Lack of systems to identify PLACI - B. Heterogeneity of PLACI - B. PLACI underreported in national databases	- Report numbers and characteristics of PLACI, with emphasis on caregivers’ absence - Develop replicable tools to identify PLACI - Flag PLACI at risk	
		- Report protocols on PLACI in national databases	
Understanding specific needs of PLACI	- B. Services often focus on physical needs	- Identify overall unmet needs - Illuminate the range of services needed	- Increased focus on cognitive, mental, and emotional needs
**2. Leveraging the diagnosis of CI**			
Unintended adverse consequences of the diagnosis of CI	- B. Loss of privileges and status following diagnosis of CI - B. A diagnosis can disqualify PLACI from services - B. A diagnosis does not qualify PLACI for LTSS	- Identify effects of diagnosis, with an equity focus - Understand whether PLACI are underdiagnosed	
Learning how to empower PLACI at the point of diagnosis and beyond	- F. Consider diagnosis as an opportunity to empower PLACI	- Identify services most useful at the point of diagnosis - International comparisons to understand best practices	- Provide wrap-around services to PLACI at the point of diagnosis
**3. Expanding and enhancing services**			
Learning how to elevate the status of home care aides	- F. Home care aides provide essential LTSS - B. Medicare does not pay for most LTSS - B. Steep costs of LTSS paid out of pocket - B. Restrictive income eligibility criteria to access Medicaid home- and community-based services	- -Provide evidence of the effect of the presence of home care aides on PLACI wellbeing - Identify priorities/concerns of home care aides and their employers - Identify mechanisms to increase home care aides’ status - Identify the most appropriate supports and services for family members and other caregivers - Understand how to honor immigrant home care aides - International comparisons to understand best practices	- Expand criteria to access publicly paid home care aides - Ensure access to qualified, affordable, and language-concordant home care aides - Increase home care aides’ standing
Restrictive income eligibility criteria to access Medicaid home and community-based services	- B. Only available to a fraction of PLACI	- -Estimate PLACI who cannot qualify for public home care aides - Elucidate effects of spending down to access LTSS - Elucidate effects of Medicaid estate recovery program	
Adapting to diverse cultural values	- F. Culturally relevant LTSS likely to increase acceptance	- Understand the features of culturally appropriate services	
Learning how to have a unique integrated point of reference to access services	- B. Information about LTSS is hard to find - B. Siloed services - B. Shortages of home care aides - B. Crisis-driven services - B. Systemic racism - B. Limited language-concordant services	- Understand the feasibility of an integrated point of reference - Understand whether and how innovative initiatives to provide one-stop point of service can be tailored to PLACI	
Learning how to incorporate technology in PLACI’s LTSS	- F. Untapped opportunities of technologies - F. Pandemic accelerated progress - B. Unsolved ethical issues	- Understand what technologies empower and support PLACI - Draw lessons from the successful use of telehealth to manage chronic conditions - Address ethical issues - Understand perspectives of diverse PLACI - Understand how to increase LTSS workers’ technological skills	

## Data Availability

Data supporting the reported results will be deposited in the Consortium for Political and Social Research.
